# Telitacicept as a potential therapeutic alternative in autoantibody-negative probable autoimmune encephalitis: a case report

**DOI:** 10.1186/s12883-026-04995-2

**Published:** 2026-05-27

**Authors:** Chaowei Xu, Yun Zhang, Jian Chen

**Affiliations:** Department of Neurology, Affiliated Jinhua Hospital, Zhejiang University School of Medicine, Jinhua, China

**Keywords:** Autoimmune encephalitis, Autoantibody-negative but probable autoimmune encephalitis, Telitacicept, Diagnosis, Treatment

## Abstract

**Background:**

The diagnosis and treatment of autoantibody-negative autoimmune encephalitis (AbNAE) remain challenging due to the absence of definitive serological markers and established treatment strategies.

**Case presentation:**

We present a case of a 54-year-old male with acute memory impairment. Cerebrospinal fluid (CSF) analysis and cranial magnetic resonance imaging (MRI) revealed features consistent with neuroinflammation. However, the autoimmune encephalitis (AE) antibody panel in both CSF and serum returned negative results. Initially misdiagnosed as viral encephalitis (VE), the patient received a three-week antiviral regimen. Subsequently, the patient developed seizures and neuropsychiatric symptoms, with concurrent deterioration on serial electroencephalography (EEG) and MRI. Repeat AE antibody testing remained negative. The diagnosis of autoantibody-negative but probable autoimmune encephalitis (ANPRA), a subtype of AbNAE, was established. Despite prompt initiation of first-line immunotherapies, including high-dose corticosteroids and intravenous immunoglobulin (IVIg), the patient’s neurological status continued to decline. Therefore, subcutaneous telitacicept (160 mg weekly) was administered for four consecutive weeks. This intervention led to progressive neurological improvement without clinically significant adverse events.

**Conclusion:**

This case highlights the aggressive nature and suboptimal response to conventional immunotherapy in ANPRA, and suggests that telitacicept may represent a viable therapeutic alternative for this challenging condition.

## Introduction

AE encompasses a spectrum of central nervous system inflammatory disorders mediated by autoimmune mechanisms [1]. A subset of patients with suspected antibody-mediated encephalitis remain negative despite comprehensive antibody screening, posing diagnostic and therapeutic challenges [2]. Therapeutic strategies for AbNAE are largely adapted from those developed for antibody-positive cases [3]. Here we report a case of ANPRA that was refractory to conventional IVIg plus corticosteroid therapy but achieved clinical stabilization after the addition of telitacicept, a dual B-cell–modulating fusion protein [4]. This observation provides novel real-world evidence supporting the efficacy of telitacicept in ANPRA and offers a valuable reference for precision management of refractory antibody-negative cases.

## Clinical report

**Fig. 1:**
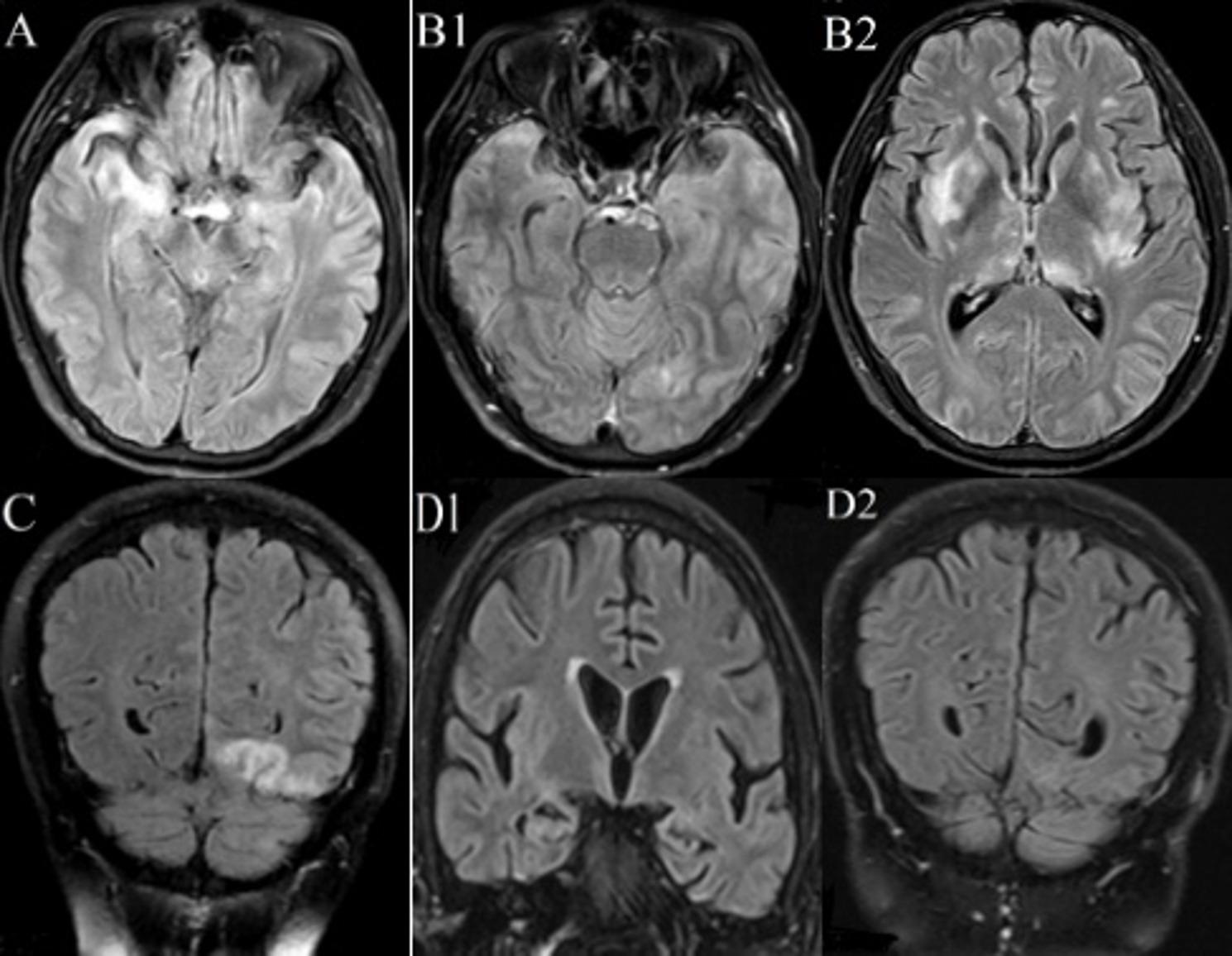
Serial cranial MR FLAIR image findings. **A** September 8, 2025: FLAIR hyperintense inflammatory lesions in the bilateral temporal lobes and hippocampi. **B** November 9, 2025: Improved hippocampal signal intensity with associated atrophy (**B1**), but increased lesion burden in the basal ganglia (**B2**). **C **November 17, 2025: New hyperintense lesions in the left occipital lobe. **D** January 27, 2026: Near-complete resolution of all previously noted intracranial lesions, including those in the basal ganglia (**D1**) and occipital regions (**D2**)

A 54-year-old man presented on 7 September 2025 with a 5-day history of acute memory impairment; he denied recent fever, diarrhoea, vaccination, or drug exposure. The deficit was characterised by persistent anterograde amnesia and spatial cognitive impairment; there were no headaches, seizures, psychosis, or systemic symptoms. He was afebrile, neck-supple, with normal limb strength, intact coordination and sensation, and absent plantar responses. Neuropsychological evaluation demonstrated selective anterograde amnesia. The patient was unable to recall any of three previously encoded items (ball, national flag, and tree) during immediate recall or following a 5-minute delay. The EEG obtained on September 8, recorded with the patient awake and eyes closed, revealed a well-organized 10–11 Hz, low-to-medium amplitude alpha rhythm that was essentially symmetrical bilaterally, indicating no significant abnormality. Cranial MRI revealed bilateral fluid-attenuated inversion recovery (FLAIR) hyperintensities confined to the medial temporal lobes and hippocampi, with a small patchy enhancing lesion in the left hippocampus, indicative of an inflammatory process (Fig. 1A). Diagnostic lumbar puncture performed the same day yielded crystal-clear CSF, the pressure was 160 mmH₂O. CSF analysis showed 87 nucleated cells/µL (98% mononuclear), positive Pandy test, protein 663 mg/L, and lactate dehydrogenase 62.4 U/L. All assays from the Hangzhou Dian Medical Laboratory Center yielded negative results: CSF metagenomic next-generation sequencing (mNGS); serum and CSF testing for AE antibodies (via CBA, including NMDAR, AMPAR1/2, LGI1, CASPR2, GABABR, DPPX, IgLON5, GlyRα1, D2R, mGluR5, mGluR1, Neurexin-3α, and GAD65); serum and CSF testing for AE antibodies via tissue-based assays (TBA) with monkey brain tissue, and serum immunoblotting for onconeural antibodies (SOX1, GAD65, Zic4, Tr, Ma1, Ma2, Amphiphysin, CV2, Ri, Yo, Hu). Immunologic tests, such as antinuclear antibodies, antineutrophil cytoplasmic antibodies, and anticardiolipin antibodies, showed no abnormalities. Extensive diagnostic workup disclosed no evidence of underlying neoplasia. Empirical intravenous acyclovir 500 mg every 8 h was commenced. Lumbar puncture on 17 September showed improving parameters (pressure 120 mmH₂O; 63 cells/µL, 99% mononuclear; protein 784 mg/L; LDH 56 U/L). Following a complete 3-week course of empirical antiviral therapy, the patient’s symptoms persisted but remained stable; however, a decline in CSF cell count was observed. The patient was discharged with a diagnosis of VE.

On 27 October 2025 the patient was emergently admitted after a witnessed generalized tonic–clonic seizure lasting approximately 3 min, characterized by loss of consciousness, four-limb jerking, trismus, and oral foaming. Post-ictally he was lethargic, bradyphrenic, and unable to cooperate with neurological examination. Cranial MRI conducted at the local hospital on the same day demonstrated multiple intracranial inflammatory lesions, but the images could not be obtained. He was transferred to our hospital the following day for continued inpatient care. EEG on 28 October showed a poorly organized background rhythm of 5.5–6.5 Hz, characterized by low-to-medium amplitude theta activity. A significant amount of 3–4 Hz medium-to-high amplitude delta and theta waves are observed diffusely across all leads, appearing as sporadic or brief-to-prolonged runs. Notably, the left temporal region exhibits frequent high-amplitude sharp-and-slow-wave complexes, which recur periodically at intervals of 1–2 s. Lumbar puncture on 30 October yielded crystal-clear CSF, the pressure was 160 mmH₂O. CSF analysis showed 17 nucleated cells/µL (71% mononuclear), positive Pandy test, protein 693 mg/L, and lactate dehydrogenase 56 U/L. Oligoclonal bands were type I, the test result of CSF and serum AE antibody panel remained negative (Hangzhou Dian Medical Laboratory Center).

In light of the subacute disease progression, evidence of deterioration on both radiological and electrophysiological evaluations, and an inflammatory CSF signature, the patient fulfilled the diagnostic criteria for ANPRA, a diagnosis of ANPRA was established [1]. Levetiracetam was initiated at 500 mg twice daily for seizure control. We initiated a five-day course of IVIg at 0.4 g/kg/day. Concurrently, intravenous methylprednisolone was started at 500 mg/day for 5 days, followed by 240 mg daily for 3 days and 120 mg daily for 3 days, then transitioned to oral prednisone 1 mg/kg/day.

Cranial MRI on 9 November showed an increased number of intracranial lesions, although the morphology of select pre-existing foci had improved (Fig. 1B1, B2). On 13 November the patient developed neuropsychiatric symptoms as incessant self-talk and physical aggression; no epileptic events were recorded. Repeat MRI on 17 November showed progression of the left occipital lesion (Fig. 1C).

Owing to suboptimal therapeutic response, telitacicept 160 mg subcutaneously was initiated on 17 November and administered weekly for four consecutive weeks. Psychotic symptoms progressively abated, independence in activities of daily living was restored, and disease control was achieved. No adverse events such as allergic reactions, infections, or leukopenia were observed during treatment with Telitacicept. At discharge, maintenance immunotherapy comprised prednisone 50 mg once daily plus mycophenolate mofetil 0.5 g twice daily. The most recent follow-up was conducted on January 27, 2026. The patient remained clinically stable, and follow-up cranial MRI demonstrated improvement in multiple intracranial lesions (Fig. 1D1, D2), confirming the final diagnosis of ANPRA.

### Discussion and conclusions

The diagnosis of AE conventionally hinges on the demonstration of neural autoantibodies and a favorable response to immunotherapy [5]. Yet a clinically indistinguishable cohort remains negative, rendering early recognition formidable. The reason for the antibody negativity can stem from pre-epitope-sparing sampling, non-standardized assays, restricted antibody panels, or exclusive testing of either serum or CSF. Accumulating evidence now positions AbNAE as a distinct and substantial phenotypic variant [6]. Diagnostic uncertainty in these cases frequently delays immunotherapy, thereby compromising neurologic outcome [7]. 

AE and VE overlap in clinical phenotype, CSF signature, and neuroimaging appearance, often resulting in diagnostic delay or misattribution [8–10]. At initial presentation, extensive autoantibody profiling of serum and CSF identified no known neuronal surface or intracellular antigens. Empirical intravenous acyclovir was administered for 3 weeks. CSF pleocytosis declined modestly, the patient’s symptoms persisted but remained stable, prompting discharge with a presumptive diagnosis of VE.

One month later, the patient experienced new-onset seizures and florid neuropsychiatric deterioration. The EEG findings demonstrated deterioration compared to the previous study. Cranial MRI showed a marked increase in the number and confluence of FLAIR-hyperintense lesions. The test result of CSF and serum AE antibody panel remained negative. The 2016 Graus criteria stratify AbNAE into two discrete phenotypes: definite autoimmune limbic encephalitis (ALB) and ANPRA [1]. The patient met published criteria for ANPRA. The negative result of mNGS of the CSF obviated a diagnosis of post‑viral‑encephalitis AE. We therefore established a diagnosis of ANPRA and initiated escalated immunotherapy.

Treatment strategies for AbNAE are largely adapted from regimens originally developed for antibody-positive cases. First-line therapy for acute AE comprises high-dose corticosteroids, IVIg, or plasma exchange; patients who fail to improve are escalated to second-line agents such as rituximab and cyclophosphamide [3]. Evidence indicates that ANPRA patients are given IVIg and second-line immunotherapies far more frequently than their ALB counterparts, highlighting both the heightened severity of ANPRA and its conspicuously blunted response to first-line therapy [11]. 

Despite prompt initiation of high-dose corticosteroids and IVIg, the patient’s neurological status continued to deteriorate. The absence of a pathognomonic antibody and the attendant diagnostic uncertainty engendered pronounced hesitation in selecting an escalated immunotherapy regimen. Previous studies have similarly demonstrated that AbNAE is associated with poorer immunotherapeutic responses, largely because the lack of a definitive biomarker delays treatment initiation and fosters more conservative therapeutic strategies [7]. 

B lymphocytes drive the onset, progression, and relapse of AE through antibody production, antigen presentation, and pro-inflammatory cytokine release, making them a key therapeutic target [12]. Telitacicept, a recombinant fusion protein that neutralizes both B-lymphocyte stimulator (BLyS) and a proliferation-inducing ligand (APRIL), suppresses aberrant B-cell activation and expansion [4]. Furthermore, long-lived plasma cells (LLPCs) do not express CD20 and are resistant to anti-CD20 monoclonal antibodies such as rituximab; however, telitacicept can effectively suppress the survival and antibody production of these LLPCs [13]. The agent has shown efficacy in diverse autoimmune disorders, and isolated reports have documented its benefit in antibody-positive AE, although evidence remains scarce [14, 15]. In summary, telitacicept demonstrates distinct clinical advantages: it broadly suppresses the upstream stages of B-cell maturation and differentiation, while effectively targeting rituximab-refractory LLPCs. Furthermore, its subcutaneous administration enhances patient convenience. Unlike cyclophosphamide—a highly potent yet toxic agent whose severe adverse effects constrain its long-term utility—telitacicept exhibits a superior safety profile.

The dosage and administration frequency of telitacicept in this patient were based on the regimen previously described for antibody-positive AE [14, 15]. After obtaining informed consent, we initiated telitacicept 160 mg subcutaneously once weekly for four consecutive weeks. The patient’s neurological status improved progressively without clinically significant adverse events. We provide the first demonstration that telitacicept can induce remission in ANPRA, expanding its therapeutic spectrum. This index case warrants validation in larger cohorts to establish telitacicept as a treatment option for antibody-negative autoimmune disease.

Here we describe a case of ANPRA that remained refractory to first-line IVIg plus high-dose corticosteroids. Subcutaneous telitacicept 160 mg weekly for four weeks was added, resulting in neurological improvement without clinically significant adverse events. This observation expands the emerging evidence that dual BLyS/APRIL blockade can induce remission in ANPRA. Telitacicept may therefore represent a valuable therapeutic option for patients who fail conventional immunotherapy; nevertheless, longer follow-up and larger prospective cohorts are required to definitively establish its efficacy and safety in this indication.

## Data Availability

All data generated or analyzed during this study are included in this published article.

## Abbreviations

AbNAE: Autoantibody-negative autoimmune encephalitis
ANPRA: Autoantibody-negative but probable autoimmune encephalitis
CSF: Cerebrospinal fluid
MRI: Magnetic resonance imaging
FLAIR: Fluid-attenuated inversion recovery
AE: Autoimmune encephalitis
VE: Viral encephalitis
EEG: Electroencephalography
IVIg: Intravenous immunoglobulin
mNGS: Metagenomic next-generation sequencing
LLPCs: Long-lived plasma cells

## References

[1] Graus F, Titulaer MJ, Balu R, et al. A clinical approach to diagnosis of autoimmune encephalitis. Lancet Neurol. 2016;15(4):391–404. 10.1016/S1474-4422(15)00401-9. 26906964 10.1016/S1474-4422(15)00401-9 PMC5066574

[2] Mangioris G, Orozco E, Dubey D, et al. Long-term outcomes in antibody-negative autoimmune encephalitis: a retrospective study. J Neurol. 2024;271(12):7502–15. 10.1007/s00415-024-12680-y. 39278895 10.1007/s00415-024-12680-y PMC12477645

[3] Abboud H, Probasco JC, Irani S, et al. Autoimmune encephalitis: proposed best practice recommendations for diagnosis and acute management. J Neurol Neurosurg Psychiatry. 2021;92(7):757–68. 10.1136/jnnp-2020-325300. 33649022 10.1136/jnnp-2020-325300 PMC8223680

[4] Liu B, Zhao Y, Liu D, Li X, Ma Z, Yang Q. The Latest Progress in the Application of Telitacicept in Autoimmune Diseases. Drug Des Devel Ther. 2024;18:5811–25. 10.2147/DDDT.S493923. 39664967 10.2147/DDDT.S493923 PMC11633291

[5] Venkatesan A, Tunkel AR, Bloch KC, et al. Case definitions, diagnostic algorithms, and priorities in encephalitis: consensus statement of the international encephalitis consortium. Clin Infect Dis. 2013;57(8):1114–28. 10.1093/cid/cit458. 23861361 10.1093/cid/cit458 PMC3783060

[6] Lee WJ, Lee HS, Kim DY, et al. Seronegative autoimmune encephalitis: clinical characteristics and factors associated with outcomes. Brain. 2022;145(10):3509–21. 10.1093/brain/awac166. 35512357 10.1093/brain/awac166

[7] Gastaldi M, Mariotto S, Giannoccaro MP, et al. Subgroup comparison according to clinical phenotype and serostatus in autoimmune encephalitis: a multicenter retrospective study. Eur J Neurol. 2020;27(4):633–43. 10.1111/ene.14139. 31814224 10.1111/ene.14139

[8] Qiao S, Zhang C, Li H, Zhou T, Wang A, Zhang S. Abnormal CSF-Specific OCBs in Neuronal Surface Antibody-Associated Autoimmune Encephalitis Differentiating from Viral Encephalitis. J Inflamm Res. 2025;18:2307–16. 10.2147/JIR.S504003. 39991667 10.2147/JIR.S504003 PMC11844203

[9] Tan Y, Liu M, He L. Clinical and MRI differential analysis of autoimmune encephalitis and viral encephalitis. J Taibah Univ Med Sci. 2023;18(2):271–8. 10.1016/j.jtumed.2022.09.016. 36817222 10.1016/j.jtumed.2022.09.016 PMC9926194

[10] Wang W, Li JM, Hu FY, et al. Anti-NMDA receptor encephalitis: clinical characteristics, predictors of outcome and the knowledge gap in southwest China. Eur J Neurol. 2016;23(3):621–9. 10.1111/ene.12911. 26563553 10.1111/ene.12911

[11] Di Cosmo L, Mulic-Al Bunni S, Goh Y, et al. Systematic Review and Meta-Analysis of the Clinical Features Associated With Seronegative Autoimmune Encephalitis. Neurol Neuroimmunol Neuroinflamm. 2026;13(2):e200540. 10.1212/NXI.0000000000200540. 41499723 10.1212/NXI.0000000000200540 PMC12794537

[12] Shang H, Shen X, Yu X, Zhang J, Jia Y, Gao F. B-cell targeted therapies in autoimmune encephalitis: mechanisms, clinical applications, and therapeutic potential. Front Immunol. 2024;15:1368275. 10.3389/fimmu.2024.1368275. 38562943 10.3389/fimmu.2024.1368275 PMC10982343

[13] Zhang E, Zhao C, Wang F, Wang L, Li S, Jiang J. Inhibition of long-lived plasma cells, a critical role for Telitacicept treatment to systemic lupus erythematosus. Immunopharmacol Immunotoxicol. 2025;47(5):579–89. 10.1080/08923973.2025.2511761. 40734465 10.1080/08923973.2025.2511761

[14] Zhang J, Hu M, Wang C, Guo S. Successful sequential therapy with rituximab and telitacicept in refractory Anti-NMDA receptor encephalitis and MOG-associated demyelination: a case report and literature review. Front Immunol. 2025;16:1509143. 10.3389/fimmu.2025.1509143. 39981240 10.3389/fimmu.2025.1509143 PMC11839722

[15] Lin Y, Diao Z, Low J, Wang A, Wu W, Cao L. Therapeutic efficacy of telitacicept in a patient with GFAP autoimmune astrocytopathy: a case report. Front Immunol. 2025;16:1691029. 10.3389/fimmu.2025.1691029. 41181139 10.3389/fimmu.2025.1691029 PMC12571838

